# OC-STAMP Overexpression Drives Lung Alveolar Epithelial Cell Type II Senescence in Silicosis

**DOI:** 10.1155/2021/4158495

**Published:** 2021-08-14

**Authors:** Tian Li, Xin-yu Yang, Ding-jie Xu, Zi-yi Gao, Yi-bing Gao, Fu-yu Jin, Ya-qian Li, Shu-peng Liu, Shi-feng Li, Xue-min Gao, Wen-chen Cai, Na Mao, Zhong-qiu Wei, He-liang Liu, Ying Sun, Fang Yang, Hong Xu

**Affiliations:** ^1^Basic Medical College, Hebei Key Laboratory for Chronic Diseases, North China University of Science and Technology, Tangshan, Hebei Province 063210, China; ^2^Traditional Chinese Medicine College, North China University of Science and Technology, Tangshan, Hebei Province 063210, China; ^3^School of Public Health, Hebei Key Laboratory for Organ Fibrosis Research, North China University of Science and Technology, Tangshan, Hebei Province 063210, China

## Abstract

Cellular senescence has been considered an important driver of many chronic lung diseases. However, the specific mechanism of cellular senescence in silicosis is still unknown. In the present study, silicotic rats and osteoclast stimulatory transmembrane protein (*Ocstamp*) overexpression of MLE-12 cells were used to explore the mechanism of OC-STAMP in cellular senescence in alveolar epithelial cell type II (AEC2). We found an increasing level of OC-STAMP in AEC2 of silicotic rats. Overexpression of *Ocstamp* in MLE-12 cells promoted epithelial-mesenchymal transition (EMT), endoplasmic reticulum (ER) stress, and cellular senescence. Myosin heavy chain 9 (MYH9) was a potential interacting protein of OC-STAMP. Knockdown of *Ocstamp* or *Myh9* inhibited cellular senescence in MLE-12 cells transfected with pcmv6-*Ocstamp*. Treatment with 4-phenylbutyrate (4-PBA) to inhibit ER stress also attenuated cellular senescence *in vitro* or *in vivo*. In conclusion, OC-STAMP promotes cellular senescence in AEC2 in silicosis.

## 1. Introduction

Silicosis is a chronic occupational lung disease caused by long-term inhalation of free crystalline silica dust and is characterized by silicotic lesions and progressive massive fibrosis [1]. Injury, loss, and disruption of alveolar epithelial cell type II (AEC2) play a central role in pulmonary fibrosis due to its critical function in alveolar niche homeostasis through the production of pulmonary surfactant and as progenitor cells to self-renew and transdifferentiate into AEC1 [2].

Cellular senescence is now considered an important driving mechanism for chronic lung diseases, particularly chronic obstructive pulmonary disease (COPD) and idiopathic pulmonary fibrosis (IPF) [3]. Cellular senescence occurs due to replicative and stress-related senescence with activation of p53 and p16^INK4a^, respectively, leading to activation of p21^CIP1^ and cell cycle arrest [3, 4]. As silicosis is an age-related and chronic occupational lung disease, silicotic patients showed significantly shorter and telomerase gene variants compared with healthy controls in response to exposure to silica [5]. Our previous study showed increasing levels of p21, cleaved caspase-3, and phosphorylated histone H_2_AX (*γ*H_2_AX) in rats exposed to inhaled silica over time [6]. We also found that silica, matrix stiffening, or their combination triggered DNA damage and replication stress in AEC2 [7]. Therefore, cellular senescence may be a key contributor to silicosis, but the exact mechanism is still unknown.

In our preliminary study, we found that the receptor activator of nuclear factor kappa-B ligand (RANKL) signaling pathway, a classic signaling pathway for regulating osteoclast differentiation, is activated in the lungs of silicotic rats, and it promoted lung inflammation and proteolytic phenotype of macrophages [8]. Interestingly, we found that the expression of osteoclast stimulatory transmembrane protein (OC-STAMP) was found in AEC2, unlike the RANKL and RANK expression in lung macrophages. Several studies have documented that OC-STAMP has an important role in cell fusion in osteoclast precursor cells and foreign body giant cells to exert function in pathogenic bone resorption [9, 10]. To date, specific knowledge about the role and regulation of OC-STAMP remains limited [11].

Furthermore, endoplasmic reticulum (ER) stress and the unfolded protein response (UPR) have been linked to lung fibrosis through regulation of AEC apoptosis, epithelial-mesenchymal transition (EMT), myofibroblast differentiation, and M2 macrophage polarization [12]. ER stress can be targeted to improve the inflammatory and cellular senescence in chronic respiratory diseases [13]. Therefore, the present study examined the molecular mechanism of ER stress and cellular senescence crosstalk regulated by OC-STAMP overexpression.

## 2. Methods

### 2.1. Silicosis Model

Wistar rats (3 w old) were employed in this study. A silicosis model was induced by inhalation of 50 ± 10 *μ*g/m^3^ of silica (s5631; Sigma-Aldrich, St. Louis, MO, USA; ground and then heated at 180°C for 6 h) for 32 w and inhalation of pure air as the control group. To inhibit ER stress, rats received 4-phenylbutyrate (4-PBA, P21005, Sigma-Aldrich) at 100 mg/Kg once daily from 24 w until 32 w [1]. All animal protocols were reviewed and approved by the Committee on the Ethics of North China University of Science and Technology (LX2019033), and they complied with the US National Institutes of Health Guide for the Care and Use of Laboratory Animals [14].

### 2.2. Cell Culture and Treatment

The MLE-12 cell line was obtained from the Chinese Academy of Sciences cell library (Shanghai, China). Cells were plated in 6 cm^2^ dishes and transfected with pCMV6-Entry (PS100001, OriGene Technologies Inc., MD, USA) and pCMV6-*Ocstamp* (MR207985, OriGene), or they were transfected with small interfering RNAs (siRNAs) targeted against *Ocstamp* and *Myh9* (RiboBio, Guangzhou, China), and treated with 50 *μ*g/mL silica or 1 mmol/L 4-PBA [1]. The target sequences of *Ocstamp*-siRNAs were CAAACGTCTTAGGCAAGT, TGGACTTCATCCTCTTCGT, and CTCAGAAGTTACCACTGT, and the target sequences of *Myh9*-siRNAs were GCTGCCAAGAAGTTGGTAT, CCATGAATTATGGGCAT, and GCAGAACATCCAGGAACTT.

### 2.3. Immunohistochemistry and Immunofluorescence Staining

Immunohistochemical staining was performed using published protocols [15] with antibodies directed against OC-STAMP (1 : 100 dilution, 2051. PB1; FabGennix Inc., Frisco, TX, USA), ABCA3 (1 : 200 dilution, ab24751, Abcam, Cambridge, UK), P21 (1 : 100 dilution, ab109520, Abcam, Cambridge, UK), *α*-smooth muscle actin (*α*-SMA, 1 : 200 dilution, ab32575, Abcam, Cambridge, UK), Proliferating Cell Nuclear Antigen (PCNA, 1 : 100 dilution, GTX100539, Genetex, Irvine, CA, USA), and Phospho-PERK (p-PERK, 1 : 100 dilution, DF7576, Affinity, Cincinnati, OH, USA) at a concentration of 1 : 200. Immunofluorescence staining was performed using published protocols with antibodies directed against ABCA3/OC-STAMP, p21/*α*-SMA, and p-PERK at a concentration of 1 : 200.

### 2.4. Western Blot

Western blot was performed using published protocols [16] with antibodies directed against OC-STAMP (2051. PB1; FabGennix Inc.), collagen type I (Col I) (ab34710, Abcam), *α*-SMA (ab32575, Abcam), E-cadherin (ab76055, Abcam), N-cadherin (ARG23870,Arigo), p-Smad2/3 (8828 s, Cell Signaling Technology, MA, USA), Smad2/3 (5678, Cell Signaling Technology), Phospho ataxia telangiectasia and Rad3-related protein (p-ATR, DF7512, Affinity), Phospho ataxia telangiectasia mutated (p-ATM, AF8410, Affinity), p-p53-S15 (AP0083, Abclonal), p21 (ab109520, Abcam), p16 (A0262, Abclonal), p-PERK (DF7576, Affinity), p-IRE1*α* (ab48187, Abcam), Phospho-nuclear factor-kappaB (p-NF-*κ*B, ARG51516, Arigo), transforming growth factor-*β*1 (TGF-*β*1, ARG56429, Arigo), TGF-*β* receptor I (TGF*β*R1, A16396, Abclonal), and TGF-*β* receptor II (TGF*β*R2, ARG59501, Arigo) at a concentration of 1 : 1000.

### 2.5. Coimmunoprecipitation (CoIP)

The interaction of OC-STAMP with Myh9 was evaluated by Co-IP. The cells were lysed in RIPA (R0020, Solarbio Life Sciences, Beijing, China) buffer containing 1% protease inhibitors. Then, 30 *μ*L of sepharose beads (FO115, Santa Cruz Biotechnology, Santa Cruz, CA, USA) and cell lysates (2 g/L) were mixed to a volume of 400 *μ*L and incubated for 2 h at 4°C on a shaker for preclearing. The clear supernatant was incubated overnight with anti-Myh9, anti-IgG antibody, and Protein A sepharose at 4°C. The beads were collected and washed 3 times with PBS before being boiled in a 2× loading buffer at 95°C for 5 min. Western blotting was used to analyse the CoIP results.

### 2.6. Real-Time Quantitative Polymerase Chain Reaction (PCR) Analysis

Reverse transcription (K1622, Thermo Scientific, USA or ZR102, ZOMANBIO, China) was performed according to company recommendations. Amplification by real-time PCR was carried out using the 2× SYBR qPCR Mix (ZF102, ZOMANBIO, China) system [17]. The sequence details were as follows: (1) Rat *Oc-stamp*: Forward: Forward: TGCTGGGCTGTGTTACTGAG, Reverse: GTGTGAAGTCGGAAGGCTGA; (2) Rat *Gapdh*, Forward: GGTGAAGGTCGGTGTGAACG, Reverse: CTCGCTCCTGGAAGATGGTG. The results were calculated via the 2^−△△CT^ method.

### 2.7. Statistical Analysis

Statistical analyses were performed using SPSS 20.0 software (IBM Corp., Armonk, NY, USA). Two-group comparisons were made using unpaired Student's *t*-test, and multiple-group comparisons were made using one-way analysis of variance followed by Tukey's post hoc test. Statistical significance was achieved when *P* < 0.05 at a 95% confidence interval.

## 3. Results

### 3.1. Silica Increases the Level of OC-STAMP in a Silicotic Model

**Figure 1:**
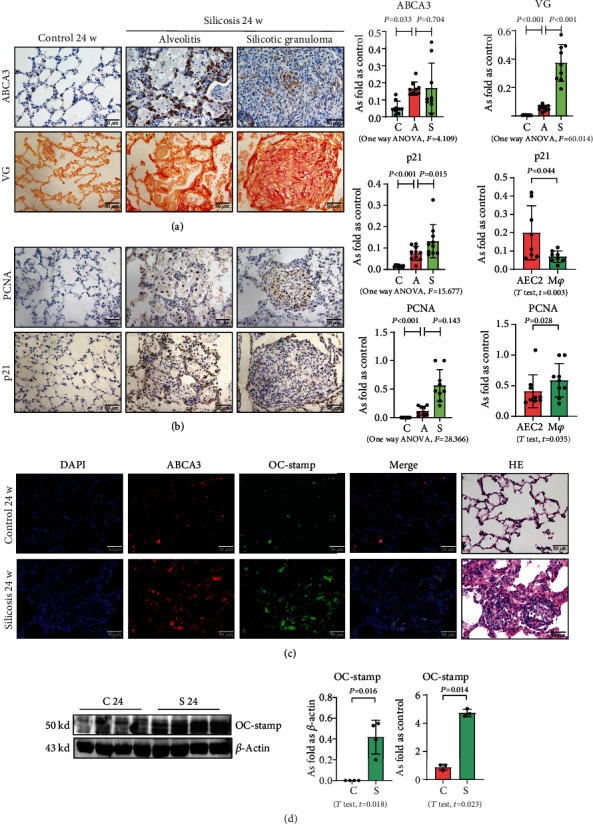
An increased level of OC-STAMP in silicotic rats. (a) Positivity of ABCA3 and collagen in silicotic rats; data are presented as the mean ± SD; *n* = 8 per group. (b) Positivity of p21 and PCNA in silicotic rats; data are presented as the mean ± SD; *n* = 8 per group. (c) Coexpression of ABCA3 and OC-STAMP in silicotic rats; (d) protein and mRNA levels of OC-STAMP in silicotic rats. Data are presented as the mean ± SD; *n* = 3 per group.

Silicotic rats were investigated in the present study, and our published reports have well documented that inhalation of silica promotes macrophage activation, myofibroblast differentiation, and collagen deposition [1, 6]. First, we used ABCA3 to identify AEC2 in silicotic rat lungs, and we found that hyperplastic AEC2 can be observed in silicotic lesions, inflammatory alveoli, and lymph nodes (Figure 1(a)). Although AEC2 in silicotic rats showed a “hyperplastic” phenotype, most of these cells showed expression of p21 but not of PCNA (Figures 1(b) and 1(c)). These results suggested that an increased level and activation of AEC2 showed a cellular senescence phenotype in rats exposed to silica. We also found coexpression of ABCA3 and OC-STAMP in silicotic rats (Figure 1(d)). The protein and mRNA levels of OC-STAMP were also increased in silicotic rats (Figure 1(d)).

### 3.2. Overexpression of OC-STAMP Promotes EMT in MLE-12 Cells

**Figure 2:**
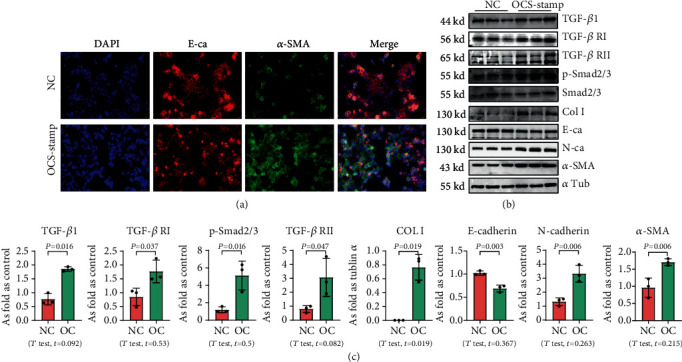
Overexpression of OC-STAMP promotes EMT in MLE-12 cells. (a) Coexpression of E-cadherin and *α*-SMA in MLE-12 cells. Bar = 50 *μ*m; (b) levels of TGF-*β*1, TGF-*β*1 R1, TGF-*β*1 R2, p-Smad2/3, Col I, E-cadherin, N-cadherin, and *α*-SMA in MLE-12 cells, measured by Western blotting. Data are presented as the mean ± SD; *n* = 3 per group.

For exploring the effect of OC-STAMP on AEC2, MLE-12 cells were transfected with the pCMV6-*Ocstamp* plasmid. As shown in Figure 2, the major profibrotic signaling pathways were measured in OC-STAMP-overexpressing cells. IF staining showed increased expression of *α*-SMA, as well as decreased expression of E-cadherin in MLE-12 cells transfected with pCMV6-*Ocstamp*. Overexpression of OC-STAMP also increased the levels of TGF-*β*1, TGF-*β*1 receptors, p-Smad, col I, N-cadherin, and *α*-SMA. The expression of E-cadherin was reduced in MLE-12 cells transfected with pCMV6-Ocstamp.

### 3.3. Overexpression of OC-STAMP Promotes ER Stress and Cellular Senescence in MLE-12 Cells

**Figure 3:**
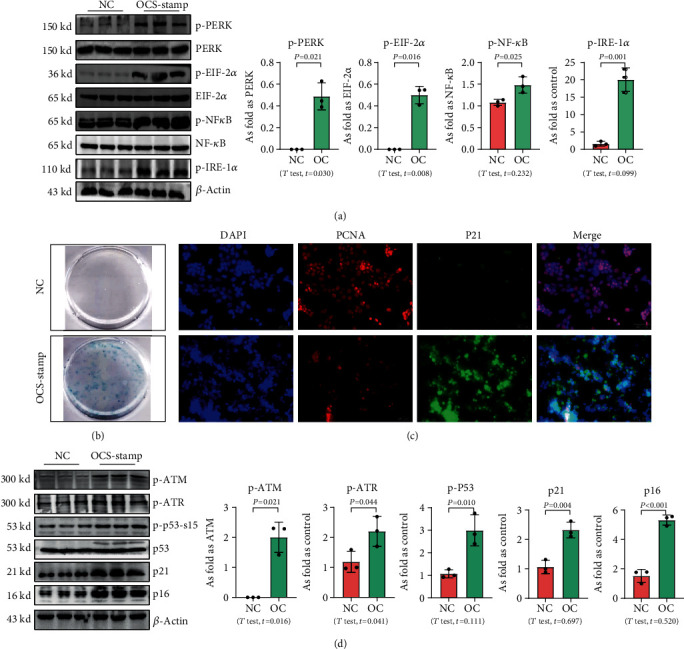
Overexpression of OC-STAMP promotes ER stress and cellular senescence in MLE-12 cells. (a) Levels of p-PERK, p-EIF 2*α*, p-NF-*κ*B, and p-IRE 1*α* in MLE-12 cells, measured by Western blotting. Data are presented as the mean ± SD; *n* = 3 per group; (b) SA-*β*-gal staining; (c) coexpression of PCNA and p21 in MLE-12 cells. Bar = 50 *μ*m; (d) levels of p-ATM, p-ATR, p-p53, p21, and p16 in MLE-12 cells measured by Western blotting. Data are presented as the mean ± SD; *n* = 3 per group.

**Figure 4:**
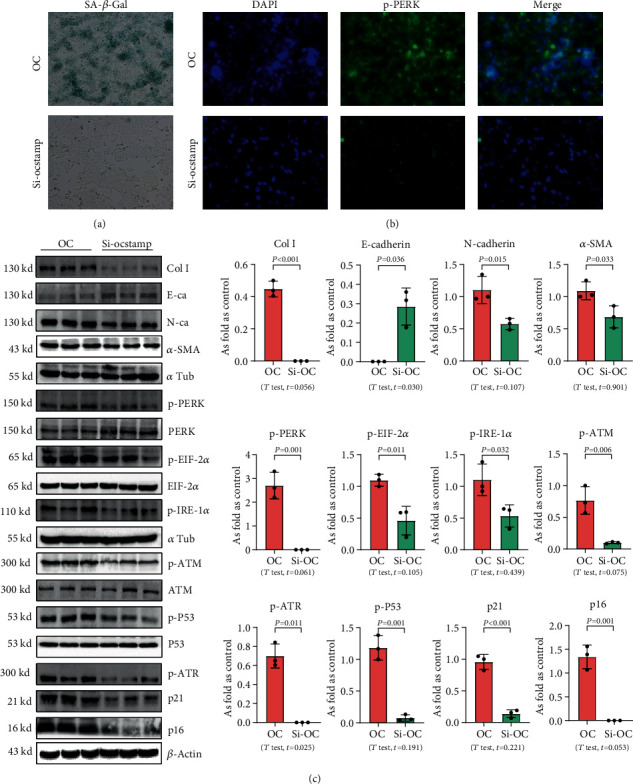
Knockdown of OC-STAMP inhibits EMT, ER stress, and cellular senescence in MLE-12 cells. (a) SA-*β*-gal staining; (b) expression of p-PERK in MLE-12 cells observed by IF staining, Bar = 50 *μ*m; (c) levels of Col I, E-cadherin, N-cadherin, *α*-SMA, p-PERK, p-EIF 2*α*, p-IRE-1*α*, p-ATM, p-ATR, p-p53, p21, and p16 in MLE-12 cells, measured by Western blotting. Data are presented as the mean ± SD; *n* = 3 per group.

Transfection with pCMV6-Ocstamp promoted ER stress in MLE-12 cells (Figure 3(a)). As shown in Figure 3(b), overexpression of OC-STAMP induced positive staining of SA-*β*-gal in MLE-12 cells. IF staining showed increased positive expression of p21 and reduced expression of PCNA in MLE-12 cells transfected with pCMV6-*Ocstamp* (Figure 3(c)). The levels of p-ATM, p-ATR, p-p53, p21, and p16 were also increased in MLE-12 cells transfected with pCMV6-*Ocstamp* (Figure 3(d)). Furthermore, silencing of *Ocstamp* inhibited cellular senescence and ER stress in MLE-12 cells transfected with *Ocstamp* (Figure 4).

### 3.4. Overexpression of OC-STAMP Induces Insensitivity in Silica-Induced MLE-12 Cells

**Figure 5:**
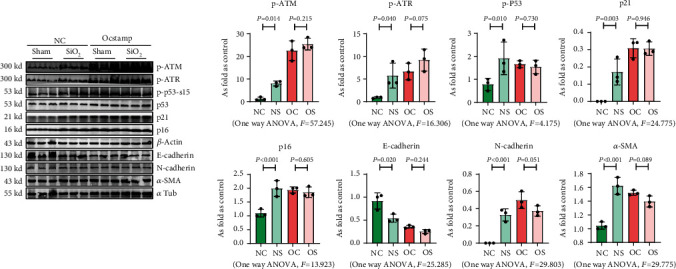
Overexpression of OC-STAMP induces insensitivity in silica-induced MLE-12 cells. Levels of p-ATM, p-ATR, p-p53, p21, p16, E-cadherin, N-cadherin, *α*-SMA in NC, and OC MLE-12 cells treated with or without silica, measured by Western blotting. Data are presented as the mean ± SD; *n* = 3 per group.

First, the cell apoptosis in MLE-12 cells treated with or without 50 *μ*g/mL silica was measured by flow cytometry; silica did not exhibit cytotoxicity in MLE-12 cells at a concentration of 50 *μ*g/mL (Figure S1). As shown in Figure 5, the pCMV6-Entry and pCMV6-*Ocstamp* cells were treated with 50 *μ*g/mL silica. Silica promoted cellular senescence in MLE-12 cells transfected with pCMV6-Entry, but it did not increase the activation of cellular senescence signaling in pCMV6-*Ocstamp* cells. Also, silica treatment did not change the EMT-related proteins in MLE-12 cells transfected with pCMV6-*Ocstamp*.

**Figure 6:**
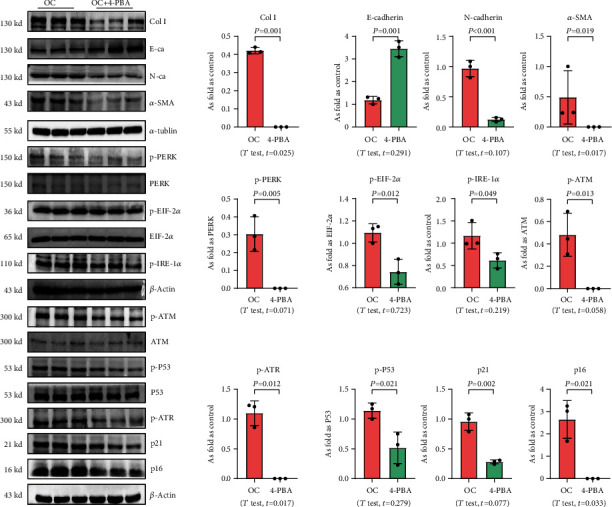
4-PBA treatment inhibits EMT, ER stress, and cellular senescence in OC-STAMP-overexpressing MLE-12 cells. Levels of Col I, E-cadherin, N-cadherin, *α*-SMA, p-PERK, p-EIF 2*α*, p-IRE-1*α*, p-ATM, p-ATR, p-p53, p21, and p16 in MLE-12 cells, measured by Western blotting. Data are presented as the mean ± SD; *n* = 3 per group.

**Figure 7:**
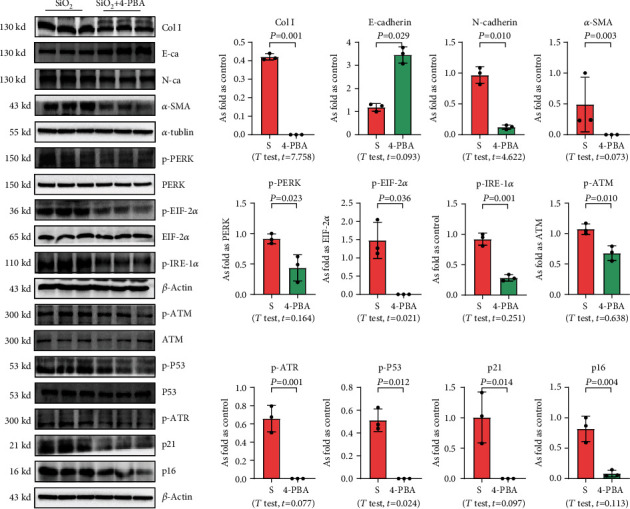
4-PBA treatment inhibits EMT, ER stress, and cellular senescence in silica-treated MLE-12 cells. Levels of Col I, E-cadherin, N-cadherin, *α*-SMA, p-PERK, p-EIF 2*α*, p-IRE-1*α*, p-ATM, p-ATR, p-p53, p21, and p16 in MLE-12 cells treated with silica, measured by Western blotting. Data are presented as the mean ± SD; *n* = 3 per group.

We used 4-PBA to explore the role of ER stress in cellular senescence induced by OC-STAMP overexpression or silica, and we found that treatment with 4-PBA inhibited the activation of senescence signaling in silica-induced or OC-STAMP overexpressing MLE-12 cells (Figures 6 and 7).

### 3.5. OC-STAMP Interacts with MYH9 to Promote Cellular Senescence

**Figure 8:**
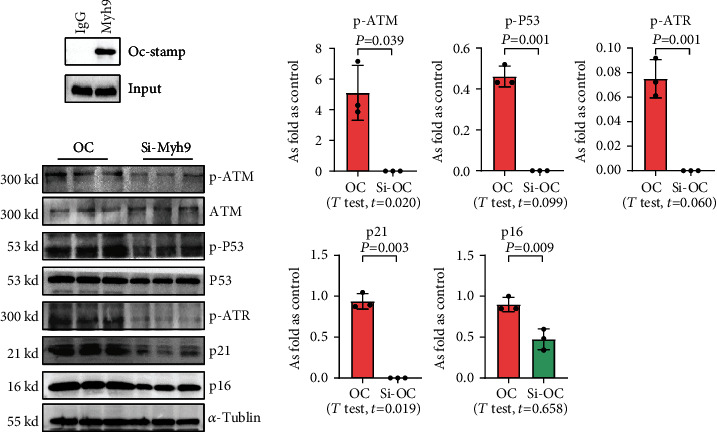
OC-STAMP interacts with MYH9. (a) Co-IP of OC-STAMP and MYH9; (b) levels of p-ATM, p-ATR, p-p53, p21, and p16 in siRNA-*Myh9* treated MLE-12 cells, measured by Western blotting. Data are presented as the mean ± SD; *n* = 3 per group.

To explore the effect of OC-STAMP in MLE-12 cells, we screened for potential OC-STAMP interacting proteins. We performed CoIP using an anti-OC-STAMP antibody followed by LC-MS/MS assay. MYH9 was identified as a potential OC-STAMP interacting protein. As shown in Figure 8, Western blot analysis of the precipitates with an OC-STAMP antibody indicated OC-STAMP CoIP with MYH9. Downregulated expression of MYH9 with siRNA also inhibited senescence signaling in *Ocstamp-*overexpressing MLE12 cells.

### 3.6. Inhibition of ER Stress Attenuates Cellular Senescence in Silicotic Rats

**Figure 9:**
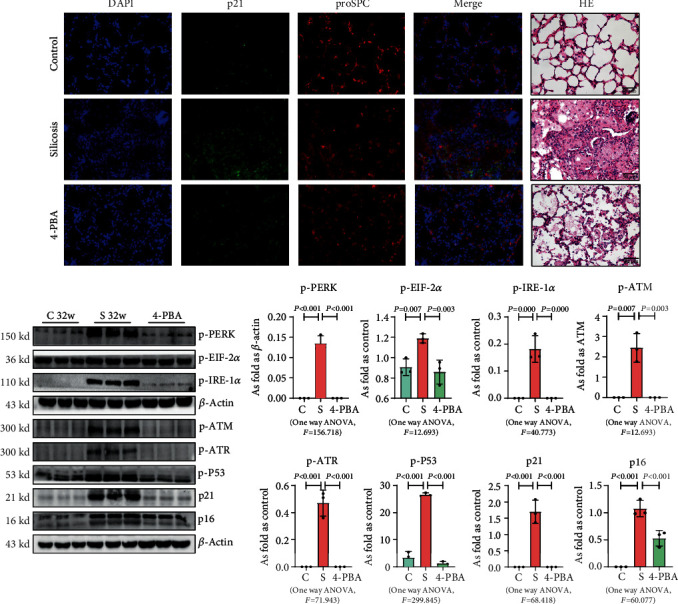
4-PBA attenuates cellular senescence in silicotic rats. (a) Coexpression of p21 and proSPC in silicotic rats, Bar = 50 *μ*m; (b) levels of p-PERK, p-EIF 2*α*, p-IRE-1*α*, p-ATM, p-ATR, p-p53, p21, and p16 in MLE-12 cells treated with silica, measured by Western blotting. Data are presented as the mean ± SD; *n* = 3 per group.

In the present study, the established model [1] was used to explain the effect of ER stress on cellular senescence in silicotic rats. Treatment with 4-PBA attenuated the activation of senescence signaling, collagen deposition, and high expression of OC-STAMP in silicotic rats, which suggested that blocking of ER stress inhibited cellular senescence in pulmonary fibrosis induced by silica (Figure 9).

## 4. Discussion

Hypertrophy and hyperplasia of AEC2 is one of the prominent features of silicosis and is consistently associated with alveolitis, but the contribution of AEC2 in the pathogenesis of silicosis is largely unknown [18, 19]. Several studies have suggested that hypertrophic and hyperplastic AEC2 was proliferative AEC2, as well as enhanced production and secretion of phospholipids and surfactant proteins for lung injury and repair [20, 21]. In the present study, we analyzed the number of AEC2, collagen deposition, expression of p21 and PCNA in normal alveoli, alveolitis (consisting of macrophages and hypertrophic AEC2), and silicotic granulomas. As observed in previous studies, hypertrophic and hyperplastic AEC2 was mostly located in the alveolitis-affected area and showed a senescent phenotype with more collagen deposition. An in vitro study also showed activation of cellular senescence signaling in silica-treated MLE-12 cells. Furthermore, ER stress markers were also observed in hypertrophic and hyperplastic AEC2, which suggested that stress-related senescent AEC2 may be a potential trigger for silicosis.

Most importantly, we found overexpression of OC-STAMP, one of the major factors of RANKL signaling, derived EMT, ER stress, and cellular senescence in MLE-12 cells, which showed some similar features in rats exposed to silica. OC-STAMP knockout (KO) mice showed normal skeleton, growth, and bone metabolic markers, and OC-STAMP-deficient cells isolated from bone marrow were able to differentiate into TRAP-positive osteoclasts under RANKL stimulation but could not fuse into multinucleated cells, which suggested the specific role of OC-STAMP in osteoclast multinucleation or cell fusion rather than osteoclast differentiation [9, 10]. Thus, several studies have proposed that OC-STAMP is involved in pathogenic bone resorption rather than normal bone metabolism [11]. Furthermore, OC-STAMP induced a phenotypic switch in macrophage polarization and suppressed the M1 proinflammatory state [22]. We have been described the potential proinflammatory effect of OC-STAMP in macrophages, as a member of the RANKL signaling pathway in silicosis [8]. In the present study, we found a different mechanism of OC-STAMP in AEC2 and promoted EMT, ER-stress, and cellular senescence in MLE-12 cells, which could be blocked by *Ocstamp*-siRNA or 4-PBA. Furthermore, ER stress has been reported to be associated with EMT and resulted in an increase in the p16 and p21 levels in lung epithelial cells in pulmonary fibrosi [23]. ER stress has been also observed in senescence induced by different stimuli and has been proposed as the consequence of senescent phenotype [24]. Combined with our previous study [1, 25], we speculated that the EMT, ER stress, and cellular senescence worked together in silicosis, at least in part, by the overexpression of OC-STAMP.

Our data showed that OC-STAMP interacted with nonmuscle myosin class II, isoform A (NM II-A, also known as MYH9), which regulated the senescent signaling pathway. MYH9 is an actin-binding molecular motor and is encoded by the *Myh9* gene, which participates in many crucial cellular processes, such as adhesion, cell migration, cytokinesis and polarization, maintenance of cell shape, and signal transduction [26]. It has been reported that MYH9 localization and filament assembly can be modulated by the interaction with S100A16 during kidney injury or TGF-*β* stimulation to promote cytoskeleton reorganization and EMT progression in renal tubulointerstitial fibrosis [27]. TGF-*β*1 increased MYH9 expression, and siRNA-mediated knockdown of *MYH9* remarkably repressed TGF-*β*1-induced lung fibroblast-to-myofibroblast differentiation [28]. Furthermore, inducible conditional knockout of Myh9 in the renal tubules of adult mice resulted in progressive kidney disease with expansion of ER tubules and activation of ER stress [29]. Our data showed that OC-STAMP interacted with MYH9, which regulated cellular senescence signaling in MLE-12 cells and may have an important role in silicosis. This study has some limitations. First, the results should be verified in clinical samples to strengthen the meaning of high expression of OC-STAMP in silicosis. Furthermore, the cross talk between ER stress and senescence in silicosis still needs to be explored to better understand age-related lung pathology and pathophysiology. Further studies are needed to consider and overcome these limitations.

In summary, we have shown that cellular senescence of AEC2 participates in silicosis formation. In the context of profibrotic insults, overexpression of OC-STAMP in MLE-12 cells exacerbates ER stress, EMT, and cellular senescence, and it may play an important role in silicosis. Blockage of ER stress protects against cellular senescence and pulmonary fibrosis in response to silica exposure.

## Data Availability

The underlying data of the study can be obtained by contacting the authors if it is reasonable.

## Supplementary Materials

Supplementary Materials Figure S1: the cell apoptosis in MLE-12 cells treated with silica or not. There was no statistically significant difference in apoptosis rate between MLE-12 cells treated with or without 50 *μ*g/mL silica.

## References

[1] Zhang L., Xu D., Li Q. (2018). _N_ -acetyl-seryl-aspartyl-lysyl-proline (Ac-SDKP) attenuates silicotic fibrosis by suppressing apoptosis of alveolar type II epithelial cells via mediation of endoplasmic reticulum stress. *Toxicology and Applied Pharmacology*.

[2] Katzen J., Beers M. F. (2020). Contributions of alveolar epithelial cell quality control to pulmonary fibrosis. *The Journal of Clinical Investigation*.

[3] Barnes P. J., Baker J., Donnelly L. E. (2019). Cellular senescence as a mechanism and target in chronic lung diseases. *American Journal of Respiratory and Critical Care Medicine*.

[4] Hamsanathan S., Alder J. K., Sellares J., Rojas M., Gurkar A. U., Mora A. L. (2019). Cellular senescence: the trojan horse in chronic lung diseases. *American Journal of Respiratory Cell and Molecular Biology*.

[5] Fan Y., Zheng C., Wu N., Li Y., Huang X., Ye Q. (2021). Telomerase gene variants and telomere shortening in patients with silicosis or asbestosis. *Occupational and Environmental Medicine*.

[6] Shifeng L., Hong X., Xue Y. (2019). Ac-SDKP increases *α*-TAT 1 and promotes the apoptosis in lung fibroblasts and epithelial cells double-stimulated with TGF-*β*1 and silica. *Toxicology and Applied Pharmacology*.

[7] Li G., Chen S., Zhang Y. (2020). Matrix stiffness regulates *α*-TAT1-mediated acetylation of *α*-tubulin and promotes silica-induced epithelial-mesenchymal transition via DNA damage. *Journal of Cell Science*.

[8] Jin F., Geng F., Xu D. (2021). Ac-SDKP attenuates activation of lung macrophages and bone osteoclasts in rats exposed to silica by inhibition of TLR4 and RANKL signaling pathways. *Journal of Inflammation Research*.

[9] Ishii T., Ruiz-Torruella M., Ikeda A. (2018). OC-STAMP promotes osteoclast fusion for pathogenic bone resorption in periodontitis via up-regulation of permissive fusogen CD9. *The FASEB Journal*.

[10] Witwicka H., Hwang S. Y., Reyes-Gutierrez P. (2015). Studies of OC-STAMP in osteoclast fusion: a new knockout mouse model, rescue of cell fusion, and transmembrane topology. *PLoS One*.

[11] Kodama J., Kaito T. (2020). Osteoclast multinucleation: review of current literature. *International Journal of Molecular Sciences*.

[12] Burman A., Tanjore H., Blackwell T. S. (2018). Endoplasmic reticulum stress in pulmonary fibrosis. *Matrix Biology*.

[13] Manevski M., Muthumalage T., Devadoss D. (2020). Cellular stress responses and dysfunctional mitochondrial-cellular senescence, and therapeutics in chronic respiratory diseases. *Redox Biology*.

[14] Worlein J. M., Baker K., Bloomsmith M., Coleman K., Koban T. L. (2011). The eighth edition of the guide for the care and use of laboratory animals (2011); implications for behavioral management. *American Journal of Primatology*.

[15] Hui Z., Dingjie X., Yuan Y. (2018). Silicosis decreases bone mineral density in rats. *Toxicology and Applied Pharmacology*.

[16] Chen Y., Xu D., Yao J. (2020). Inhibition of miR-155-5p Exerts Anti-Fibrotic Effects in Silicotic Mice by Regulating Meprin *α*. *Molecular Therapy - Nucleic Acids*.

[17] Gao X., Xu D., Li S. (2020). Pulmonary silicosis alters microRNA expression in rat lung and miR-411-3p exerts anti-fibrotic effects by inhibiting MRTF-A/SRF signaling. *Molecular Therapy - Nucleic Acids*.

[18] Lesur O., Cantin A. M., Tanswell A. K., Melloni B., Beaulieu J. F., Begin R. (1992). Silica exposure induces cytotoxicity and proliferative activity of type II pneumocytes. *Experimental Lung Research*.

[19] Porter D. W., Hubbs A. F., Mercer R. (2004). Progression of lung inflammation and damage in rats after cessation of silica inhalation. *Toxicological Sciences*.

[20] Miller B. E., Dethloff L. A., Hook G. E. (1986). Silica-induced hypertrophy of type II cells in the lungs of rats. *Laboratory Investigation; A Journal of Technical Methods and Pathology*.

[21] Panos R. J., Suwabe A., Leslie C. C., Mason R. J. (1990). Hypertrophic alveolar type II cells from silica-treated rats are committed to DNA synthesis in vitro. *American Journal of Respiratory Cell and Molecular Biology*.

[22] Yuan H., He J., Zhang G., Zhang D., Kong X., Chen F. (2017). Osteoclast stimulatory transmembrane protein induces a phenotypic switch in macrophage polarization suppressing an M1 pro-inflammatory state. *Acta Biochimica et Biophysica Sinica*.

[23] Phan T. H. G., Paliogiannis P., Nasrallah G. K. (2021). Emerging cellular and molecular determinants of idiopathic pulmonary fibrosis. *Cellular and Molecular Life Sciences: CMLS*.

[24] Parimon T., Hohmann M. S., Yao C. (2021). Cellular senescence: pathogenic mechanisms in lung fibrosis. *International Journal of Molecular Sciences*.

[25] Li S., Li Y., Xu H. (2020). ACE2 attenuates epithelial-mesenchymal transition in MLE-12 cells induced by silica. *Drug Design, Development and Therapy*.

[26] Asensio-Juarez G., Llorente-Gonzalez C., Vicente-Manzanares M. (2020). Linking the landscape of MYH9-related diseases to the molecular mechanisms that control non-muscle myosin II-A function in cells. *Cells*.

[27] Sun H., Zhao A., Li M. (2020). Interaction of calcium binding protein S100A16 with myosin-9 promotes cytoskeleton reorganization in renal tubulointerstitial fibrosis. *Cell Death & Disease*.

[28] Sun X., Zhu M., Chen X., Jiang X. (2021). MYH9 inhibition suppresses TGF-*β*1-stimulated lung fibroblast-to-myofibroblast differentiation. *Frontiers in Pharmacology*.

[29] Otterpohl K. L., Busselman B. W., Ratnayake I. (2020). Conditional Myh9 and Myh10 inactivation in adult mouse renal epithelium results in progressive kidney disease. *JCI Insight*.

