# Supplementary material for: OC-STAMP Overexpression Drives Lung Alveolar Epithelial Cell Type II Senescence in Silicosis
Source: Oxid Med Cell Longev. 2021 Aug 14;2021:4158495. doi: 10.1155/2021/4158495 (PMC8380176; doi:10.1155/2021/4158495)
Supplement: Supplementary Materials — Figure S1: the cell apoptosis in MLE-12 cells treated with silica or not. There was no statistically significant difference in apoptosis rate between MLE-12 cells treated with or without 50 μg/mL silica. [file 4158495.f1.docx]

**Supplementary Materials**


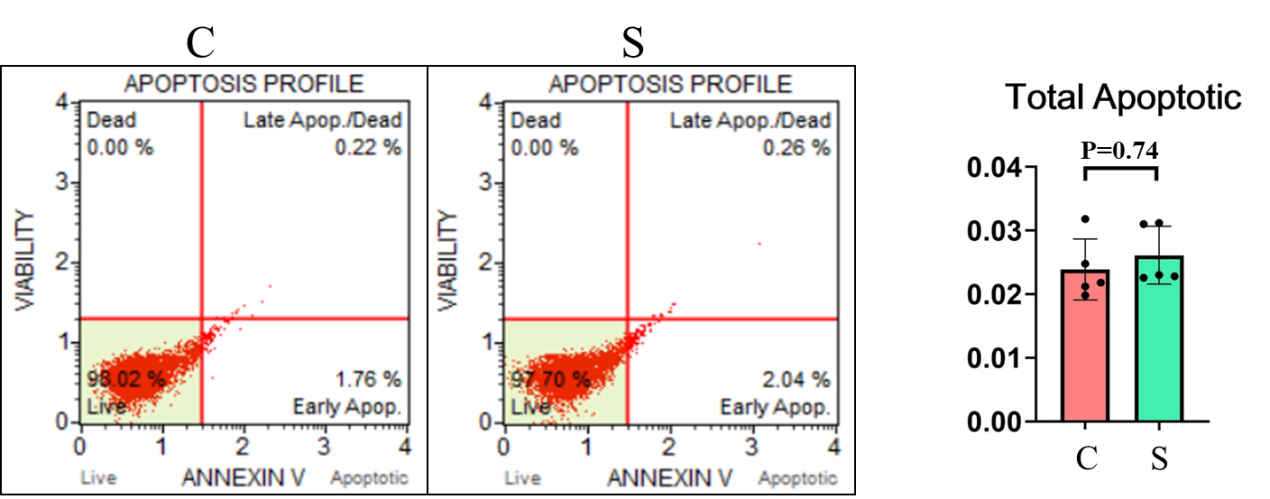


Figure S1: The cell apoptosis in MLE-12 cells treated with silica or not.

There was no statistically significant difference in apoptosis rate between MLE-12 cells treated with or without 50μg/mL silica.
